# Treatment by Organic Extracts: A Fatal Case

**Published:** 1915-05-01

**Authors:** 


### TREATMENT BY ORGANIC EXTRACTS.

### A Fatal Case.

The recent Coroner's inquiry dealing with a case in which death followed the hypodermic administration of a series of doses of one of the organic extracts is an event which has more than a local and personal interest. Opinions may possibly differ in relation to the interpretation to be given to some of the evidence, and in regard also to the opinion of the Coroner and the verdict of the jury. But just now we do not propose to challenge any one of these—at least in so far as the main issue of the inquiry is concerned. We accept for our present purpose the decision that death was due to septic poisoning; also the implication, contained in the jury's rider, that the infection was consequent on imperfect preparation of the organic extract. Of the melancholy event itself it is sufficient here to say that hardly any history can be more tragically sad than one in which measures applied with a curative intention prove in experience to be responsible for a fatal issue. The contrast between expectation and realisation could hardly be presented in a more pathetic and more poignant form.

The first point we note for comment is found in the evidence of one of the expert witnesses, who, after examination of some specimens of the extract which had been employed, declared that these specimens were not sterile. Now, these specimens, and the original package of the preparation employed, bore the name of a well-known manufacturing pharmacist. They may fairly be said, therefore, to have carried his personal guarantee both of their safety and of their efficacy. In these circumstances it is important to ask what is the real extent and value of this guarantee? Has it really a personal quality, or is it a mere indication that the manufacture was carried on in premises in his occupation and by some person whom he employed? It has always seemed to us an advisable policy, in the public interest, to encourage personal rather than company pharmacy, for the reason that in the former skill and care were constantly sharpened by a sense of individual responsibility. But to claim this position personal pharmacy must really be what it professes to be. Was it so in this case? All that the reports of the evidence tell us is that the preparation of the organic extract was effected by a "laboratory manager," while the pharmacist himself adds the assurance that never before have the extracts which carry his name been suspected as causes of septic poisoning. The question is more than a personal one. It applies over a field in which great activity, under the influence of a modern therapeutic fashion, is being displayed. The recent inquest raises the issue in a highly acute form, but it records no decision which in the least relieves the situation. Neither physicians nor pharmacists can afford to leave the question where it now stands, and the public, reasonably, will wish to know what steps are to be taken to prevent the possibility of further disasters. A personal guarantee which does not involve a personal liability seems to us to be wanting in the sense of reality.

It is satisfactory to know that the investigation acquitted the medical practitioners concerned of all suspicion of want of skill or care. At first there seemed to be some suggestions in the opposite direction, and in these circumstances it is well that the matter was thoroughly probed. Such an experience ought to be an impressive one for all practitioners who undertake methods of treatment involving the introduction of organic fluids by means of the hypodermic syringe. Such methods just now enjoy a considerable vogue, and the rarity of unhappy consequences is a tribute to the care with which the practice is followed, as it is, indeed, to the care which is taken in the processes of the laboratory. At the same time one has to be on the watch lest familiarity breed contempt. Every such administration is in essence a surgical operation, and requires the conditions which alone can guarantee safety. To the patient who has had experience the whole matter seems to be nothing more than a prick with a needle, but the practitioner knows of the risks which lurk at hand. Our reference is not in the least to the value or danger of vaccines, serums, and so on. It is merely to the method by which these are administered, and our desire is to emphasise the need for constant watchfulness lest a proceeding which is so easily accomplished be taken too lightly.

Lastly, we wish to enter a protest against a doctrine credited to one of the medical witnesses. This gentleman is reported to have said that it is the duty of a medical practitioner to carry out the directions of a specialist. By the latter term the witness presumably meant a consulting physician or surgeon; and taking this for granted, we say unhesitatingly that it is not the duty of a practitioner to carry out any scheme of treatment unless the proposal commends itself to his own judgment. No doubt he can, if he likes so to do, explain to the patient that he will, if the patient wishes it, carry out certain directions as recommended by another adviser, but certainly no duty lies on him to accept such a position. If by mutual arrangement such a position is accepted, it ought to be made clear in advance where the responsibility lies. But the position of a medical practitioner is never one in which authority is imposed on him by a superior voice. He is free to form his own judgment and to decide his own actions, and all that can be asked of him is that he shall accept responsibility for his decisions. The notion that in some superior region there dwells a race of specialists or professional supermen to whose directions the lesser lights must necessarily bow is foreign alike to the best traditions and to the liberal atmosphere of medical life. We are sure, too, that it ought to be repudiated in the interests of the public. Perhaps its enunciation during the recent inquest may show where certain ambitions tend. That these have any chance of realisation we do not for a moment believe, but it is just as well to nail them to the counter as soon as ever they betray themselves.

